# Immune patterns of the tumor microenvironment in PD-L1^+^ EGFR^+^ HNSCC patients received anti-PD-1 and EGFR-based neoadjuvant therapy

**DOI:** 10.1016/j.gendis.2025.101902

**Published:** 2025-10-24

**Authors:** Yuan Zhi, Wenhao Ren, Shaoming Li, Jingjing Zheng, Jianzhong Song, Ling Gao, Keqian Zhi

**Affiliations:** a Department of Oral and Maxillofacial Reconstruction, The Affiliated Hospital of Qingdao University, Qingdao, Shandong 266555, China; b Key Lab of Oral Clinical Medicine, The Affiliated Hospital of Qingdao University, Qingdao, Shandong 266555, China; c Department of Oral and Maxillofacial Surgery, Peking University School and Hospital of Stomatology, Beijing 100083, China; d Department of Oral and Maxillofacial Surgery, People's Hospital of Rizhao, Rizhao, Shandong 276827, China

Anti-programmed death 1 (PD-1) and epidermal growth factor receptor (EGFR)-based neoadjuvant therapy was confirmed to be effective for the treatment of head and neck squamous cell carcinoma (HNSCC). However, a previous study found that nearly 1/3 of HPV-negative HNSCC experienced no significant reduction or even increase in tumor size.^1^ Up till now, few studies have demonstrated the clinicopathological patterns of PD-L1^+^ EGFR^+^ HNSCC patients. Therefore, single-cell RNA-sequencing (scRNA-seq) analysis was applied in our retrospective study of 1077 HPV-negative HNSCC patients to characterize the immune patterns of the tumor microenvironment (TME) after anti-PD-1 and EGFR-based neoadjuvant therapy. In this study, we identified for the first time that most PD-L1^+^ EGFR^+^ HNSCC patients were in an advanced clinical stage and suffered lymph node metastasis. HPV-negative HNSCC patients experienced a significant reduction of tumor size after receiving 1 course of anti-PD-1 and EGFR-based neoadjuvant therapy. Meanwhile, PD-L1^+^ EGFR^+^ HNSCC patients have stronger invasive ability. The CXCL11/CXCR3 pair in PD-L1^+^ EGFR^+^ HNSCC cells contributed to the construction of an immunosuppressive TME by recruiting plasmacytoid dendritic cells, regulatory T cells, dendritic cells and CD8^+^ T cells. CD73, OX40, and TIM-3 are potential targets for immunotherapy sensitization in HPV-negative HNSCC. Our finding provides an immunotherapeutic sensitization strategy for PD-L1^+^ EGFR^+^ HNSCC.

Immune checkpoint blockade (ICB) is a successful immunotherapy strategy applying in HNSCC. Currently, combination immunotherapy has become a new strategy for the improvement of efficacy. Anti-PD-1 and EGFR-combined immunotherapy obtained a relatively high objective response rate (ORR) of 45% in HNSCC patients.^1^ However, nearly 1/3 of HPV-negative HNSCC experienced no significant reduction or even increase in tumor size. Hence, we first collected 1,077 HNSCC patients between May 2014 and May 2024 and divided the cohort into a PD-L1^+^ EGFR^+^ group (*n* = 882) and a non-PD-L1^+^ EGFR^+^ group (*n* = 195), which included PD-L1^+^ EGFR^−^ (*n* = 117), PD-L1^−^ EGFR^+^ (*n* = 57) and PD-L1^−^ EGFR^−^ groups (*n* = 21) (Table S1). The number of PD-L1^+^ EGFR^+^ HNSCC in the T2 stage (*n* = 330, 37.4%) (*P* < 0.0001), N1-3 stage (*n* = 312, 35.4%) (*P* = 0.0324) and clinical stage III were higher than those in the non-PD-L1^+^ EGFR^+^ HNSCC group (Fig. 1A; Table S2). Compared with those combined positive score (CPS) in 1–20, the degree of lymph node metastasis was significantly reduced in PD-L1^+^ EGFR^+^ HNSCC patients with CPS scores higher than 20 (Fig. 1B; Table S3). To investigate the relationship between PD-L1 and EGFR levels and the prognosis of HNSCC patients, we collected follow-up information of 1077 HNSCC patients for prognosis analysis. There was no significant difference in recurrence-free survival (RFS) (*P* = 0.8711) or overall survival (OAS) (*P* = 0.6620) between PD-L1^+^ EGFR^+^ and non-PD-L1^+^ EGFR^+^ HNSCC patients, which was consistent with the prognostic results of 394 HNSCC samples from The Cancer Genome Atlas (TCGA) (Fig. 1C–E). We inferred that PD-L1 or EGFR expression levels play a limited role in predicting the prognosis of HNSCC patients. Twenty-seven HNSCC patients in our cohort received a course of anti-PD-1 (Sintilimab, 200 mg) and EGFR (Nimotuzumab, 200 mg)-based neoadjuvant therapy, 15 of whom were in the PD-L1^+^ EGFR^+^ group and 12 in the non-PD-L1^+^ EGFR^+^ group. By reviewing the enhanced CT and clinical information (Fig. S1A), we found that HNSCC patients harvested a significant reduction of tumor size after receiving 1 course of neoadjuvant therapy. Although there was no statistically significant difference, PD-L1^+^ EGFR^+^ HNSCC patients exhibited better outcomes after receiving anti-PD-1 and EGFR-based combination therapy (Fig. S1B).

**Figure 1:**
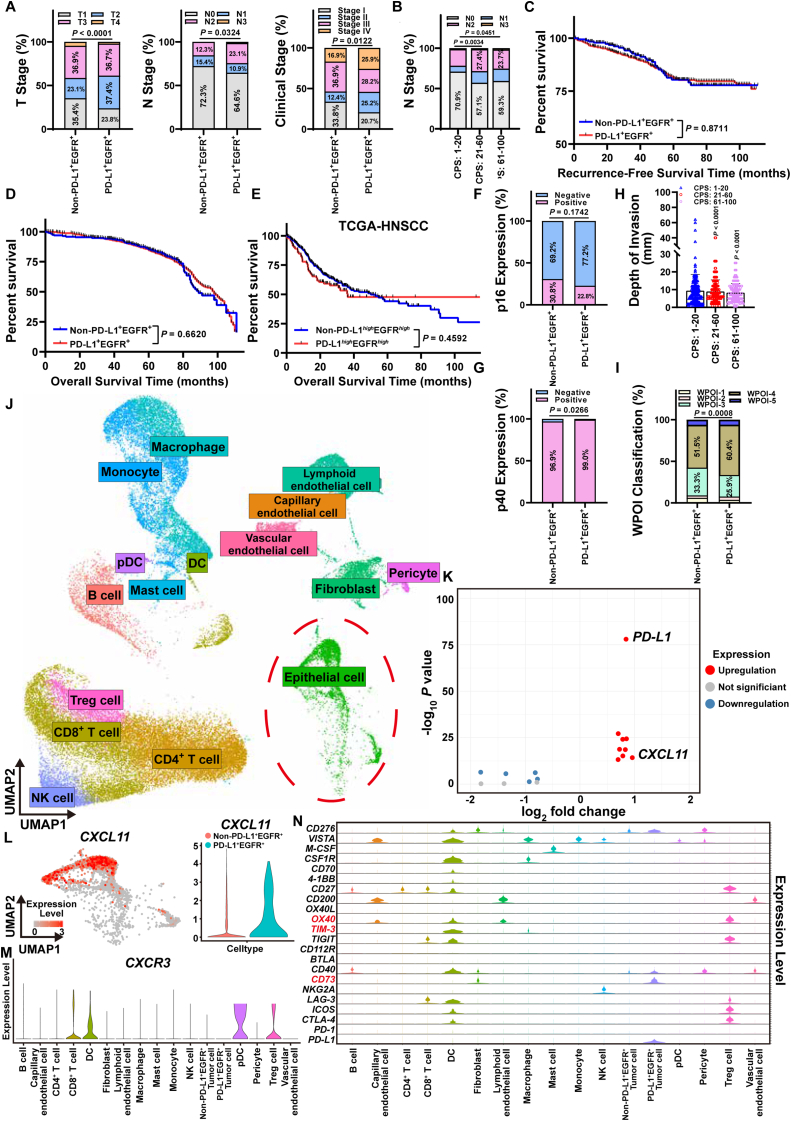
Immune patterns of the TME in PD-L1^+^ EGFR^+^ HNSCC. **(A)** Proportion of T stage (left), N stage (middle) and clinical stage (right) between PD-L1^+^ EGFR^+^ and non-PD-L1^+^ EGFR^+^ HNSCC patients. **(B)** Proportion of N stage among the three subgroups of PD-L1^+^ EGFR^+^ HNSCC patients based on the CPS. **(C)** Kaplan–Meier curves exhibiting the recurrence-free survival of PD-L1^+^ EGFR^+^ (red) and non-PD-L1^+^ EGFR^+^ (blue) HNSCC patients in our cohort. **(D)** Kaplan–Meier curves exhibiting the overall survival of PD-L1^+^ EGFR^+^ (red) and non-PD-L1^+^ EGFR^+^ (blue) HNSCC patients in our cohort. **(E)** Kaplan–Meier curves exhibiting the overall survival of PD-L1^+^ EGFR^+^ (red) and non-PD-L1^+^ EGFR^+^ (blue) HNSCC patients in HNSCC samples from TCGA database. **(F)** Proportion of p16 expression between PD-L1^+^ EGFR^+^ and non-PD-L1^+^ EGFR^+^ HNSCC patients. **(G)** Proportion of p40 expression between PD-L1^+^ EGFR^+^ and non-PD-L1^+^ EGFR^+^ HNSCC patients. **(H)** Levels of DOI among the three subgroups of PD-L1^+^ EGFR^+^ HNSCC patients based on the CPS. DOI: depth of invasion. **(I)** Proportion of WPOI classification between PD-L1^+^ EGFR^+^ and non-PD-L1^+^ EGFR^+^ HNSCC patients. WPOI: worst pattern of invasion. **(J)** Unsupervised clustering analysis (UMAP) and cell type annotation exhibiting 16 types of cells in the public scRNA-seq data (GSE164690) of HPV-negative HNSCC. Epithelial cells in the dotted circle (green) refer to tumor cells. **(K)** Volcano plot showing the DEGs between PD-L1^+^ EGFR^+^ and non-PD-L1^+^ EGFR^+^ tumor cells. Blue: down-regulated DEGs, red: up-regulated DEGs, grey: DEGs without statistical significance. **(L)** Feature plot (left) revealing the expression level of *CXCL11* in HPV-negative HNSCC. Violin plot (right) for the expression level of *CXCL11* between PD-L1^+^ EGFR^+^ and non-PD-L1^+^ EGFR^+^ tumor cells. Blue: PD-L1^+^ EGFR^+^ tumor cells, red: non-PD-L1^+^ EGFR^+^ tumor cells. **(M)** Violin plot exhibiting the expression level of *CXCR3* in HNSCC cells. **(N)** Violin plots exhibiting the expression levels of 22 immune checkpoints among cells in the TME of HPV-negative HNSCC. OX40, TIM-3 and CD73 (red) are considered potential targets for the sensitization of immune therapy in HNSCC.

Furthermore, we determined to explore the molecular reasons for the unsatisfactory efficacy of anti-PD-1 and EGFR-based neoadjuvant therapy in PD-L1^+^ EGFR^+^ HNSCC patients. The majority of PD-L1^+^ EGFR^+^ HNSCC patients were negative for p16 (77.8%) and positive for p40 (99.0%) (Fig. 1F and G). And 87.2% of HNSCC patients in our cohort were positive for EGFR expression. There was no significant difference in the DOI between the PD-L1^+^ EGFR^+^ group and the non-PD-L1^+^ EGFR^+^ group (*P* = 0.9221) (Fig. S2F). Additionally, the DOI of PD-L1^+^ EGFR^+^ HNSCC decreased as the CPS score increased (Fig. 1H). The worst pattern of invasion (WPOI) is a histopathology-based assessment of the invasive characteristics at the leading edge of solid tumors. PD-L1^+^ EGFR^+^ HNSCC had a higher WPOI-4 subtype (60.4%), whereas non-PD-L1^+^ EGFR^+^ HNSCC was predominantly the WPOI-3 subtype (33.3%), suggesting that PD-L1^+^ EGFR^+^ HNSCC has a stronger invasive ability (Fig. 1I; Fig. S2H).

To further explore the TME landscape of PD-L1^+^ EGFR^+^ HNSCC, we analyzed a public scRNA-seq data of HNSCC (GSE164690). Epithelial cells were extracted and reannotated into PD-L1^+^ EGFR^+^ tumor cells and non-PD-L1^+^ EGFR^+^ tumor cells based on the levels of PD-L1 and EGFR expression (Fig. 1J; Fig. S3A and S3B). By performing differentially expressed gene (DEG) analysis between the two types of tumor cells, we identified 9 up-regulated DEGs and 4 down-regulated DEGs (Table S4), with *CXCL11* being the most significantly up-regulated DEG (Fig. 1K). CXCL11, predominantly expressed in PD-L1^+^ EGFR^+^ tumor cells, was significantly higher than that in non-PD-L1^+^ EGFR^+^ tumor cells (Fig. 1L; Fig. S3D–S3F). The chemokine C-X-C motif receptor 3 (CXCR3) is known as a receptor of CXCL11 and plays a vital role in regulating the migration and activation of immune cells in the TME by responding to the recruitment of CXCL11. We found that *CXCR3* was mainly expressed in the plasmacytoid dendritic cells (pDCs), regulatory T (Treg) cells, dendritic cells (DCs) and CD8^+^ T cells in HNSCC, the former two of which are key immunosuppressive cells constituting the TME (Fig. 1M). Meanwhile, *PD-1* was highly expressed in CD8^+^ T cells and Treg cells, but was expressed at a lower level in pDCs sand DCs (Fig. S3C). In addition, the up-regulated guanylate binding protein (GBP1) is associated with the IFN-γ-induced immune activation.^2^ The overexpression of tryptophanyl-tRNA synthetase (*WARS*) can decrease the level of PD-1 on the surface of CD8+ T cells.^3^ *CTSC*, a well-known apoptosis-associated gene, may act as a driver of apoptosis in tumor cells.^4^ These DEGs discussed above can be regarded as the potential response to anti-tumor immunity in PD-L1^+^ EGFR^+^ tumor cells. However, there are still several significant DEGs (i.e., *F3*, *TGFBI*) associated with resistance to immunotherapy.^5^ In summary, PD-L1^+^ EGFR^+^ tumor cells recruit immunosuppressive cells such as pDCs and Treg cells through the *CXCL11*/*CXCR3* ligand–receptor pair to constitute an immunosuppressive microenvironment of HNSCC. Besides, CD8^+^ T cells and DCs are fundamental for sensitizing PD-L1^+^ EGFR^+^ HNSCC to anti-PD-1 and EGFR-based combination immunotherapy.

According to studies of ICIs in solid tumors, we summarized all immune checkpoints for which ICIs have been developed (Fig. S4). In addition to *PD-L1*, *CD73* was specifically expressed on PD-L1^+^ EGFR^+^ tumor cells, which are potential targets for immunotherapy, such as Oleclumab, JAB-BX102, and Mupadolimab, to inhibit immune evasion in HPV-negative HNSCC. The immunostimulatory molecule, *OX40*, was specifically up-regulated on Treg cells. Tumor immunotherapy sensitization can be achieved by using monoclonal antibodies to specifically block the immunostimulatory molecule *OX40* (e.g., Rocatinlimab) to inhibit Treg cell activation. Since TIM-3 is specifically up-regulated in HNSCC, Sabatolimab, a monoclonal antibody targeting TIM-3, is more likely to restrain DC-mediated anti-tumor immunity by blocking TIM-3 (Fig. 1I).

Our study, as the first study to investigate the clinical features, pathological characteristics and TME landscapes of PD-L1^+^ EGFR^+^ HNSCC, identified the clinical characteristics of most PD-L1^+^ EGFR^+^ HNSCC patients with advanced clinical stage and lymph node metastasis status for the first time. Meanwhile, PD-L1^+^ EGFR^+^ HNSCC possesses a more aggressive invasive ability. Based on the scRNA-seq analysis of HPV-negative HNSCC, the CXCL11/CXCR3 ligand-receptor pair was further recognized as a key factor in the construction of an immunosuppressive TME recruited by PD-L1^+^ EGFR^+^ HNSCC cells. In addition, CD73, OX40, and TIM-3 may become essential targets for immunotherapy sensitization in HPV-negative HNSCC. Our finding provides a fundamental mechanism for immunotherapeutic failure in HPV-negative HNSCC and an immunotherapeutic sensitization strategy for PD-L1^+^ EGFR^+^ HNSCC. Although the above finding holds promise for tumor immunotherapy, future studies based on anti-PD-1 and EGFR-based combination immunotherapy should still be conducted in real-world clinical trials to evaluate its efficacy in PD-L1^+^ EGFR^+^ HNSCC.

## CRediT authorship contribution statement

**Yuan Zhi:** Writing – original draft, Methodology, Investigation, Data curation. **Wenhao Ren:** Validation, Project administration, Investigation, Funding acquisition. **Shaoming Li:** Resources, Methodology, Formal analysis, Data curation. **Jingjing Zheng:** Project administration, Investigation, Data curation. **Jianzhong Song:** Supervision, Investigation, Data curation. **Ling Gao:** Writing – review & editing, Project administration, Conceptualization. **Keqian Zhi:** Writing – review & editing, Supervision, Project administration, Funding acquisition, Conceptualization.

## Ethics declaration

This study was approved by the Ethical Committee of the Affiliated Hospital of Qingdao University (No. AHQU-MAL20210604) to the Department of Oral and Maxillofacial Reconstruction, the Affiliated Hospital of Qingdao University, Shandong, China.

## Funding

This study was funded by the 10.13039/501100001809National Natural Science Foundation of China (No. 42176096 to L.G., No. 42176097 and 42576118 to K.Q.Z.), the 10.13039/501100007129Natural Science Foundation of Shandong Province (No. ZR2021MD065 to K.Q.Z., No. ZR2021MH305 to J.J.Z., No. ZR2022MH223 to W.H.R.), the TaiShan Scholars Foundation of Shandong Province (No. tsqn202306397 to L.G.), the State Administration of Traditional Chinese Medicine Science and Technology Department Co-construction of Science and Technology Project (No. GZY-KJS-SD-2023-078 to K.Q.Z.), the Shandong Province Medical Health Science and Technology Project (No. 202308021296 to K.Q.Z.), the Shandong Province Medical Health Science and Technology Development Plan Project (No. 202208020829 to S.M.L.), the Rizhao Traditional Chinese Medicine Science and Technology Project (No. RZY2023C02 to J.Z.S.), and the Rizhao City Natural Science Foundation Project (No. RZ2022ZR35 to J.Z.S.).

## Conflict of interests

The authors declare that there are no competing interests in this study.
