# Supplementary material for: Immune patterns of the tumor microenvironment in PD-L1+ EGFR+ HNSCC patients received anti-PD-1 and EGFR-based neoadjuvant therapy
Source: Genes Dis. 2025 Oct 24;13(4):101902. doi: 10.1016/j.gendis.2025.101902 (PMC12989833; doi:10.1016/j.gendis.2025.101902)
Supplement: Multimedia component 1 [file mmc1.pdf]

Fig. S1 Anti-PD-1 and EGFR-based Neoadjuvant Therapy Significantly Reduces Tumor Size of HNSCC

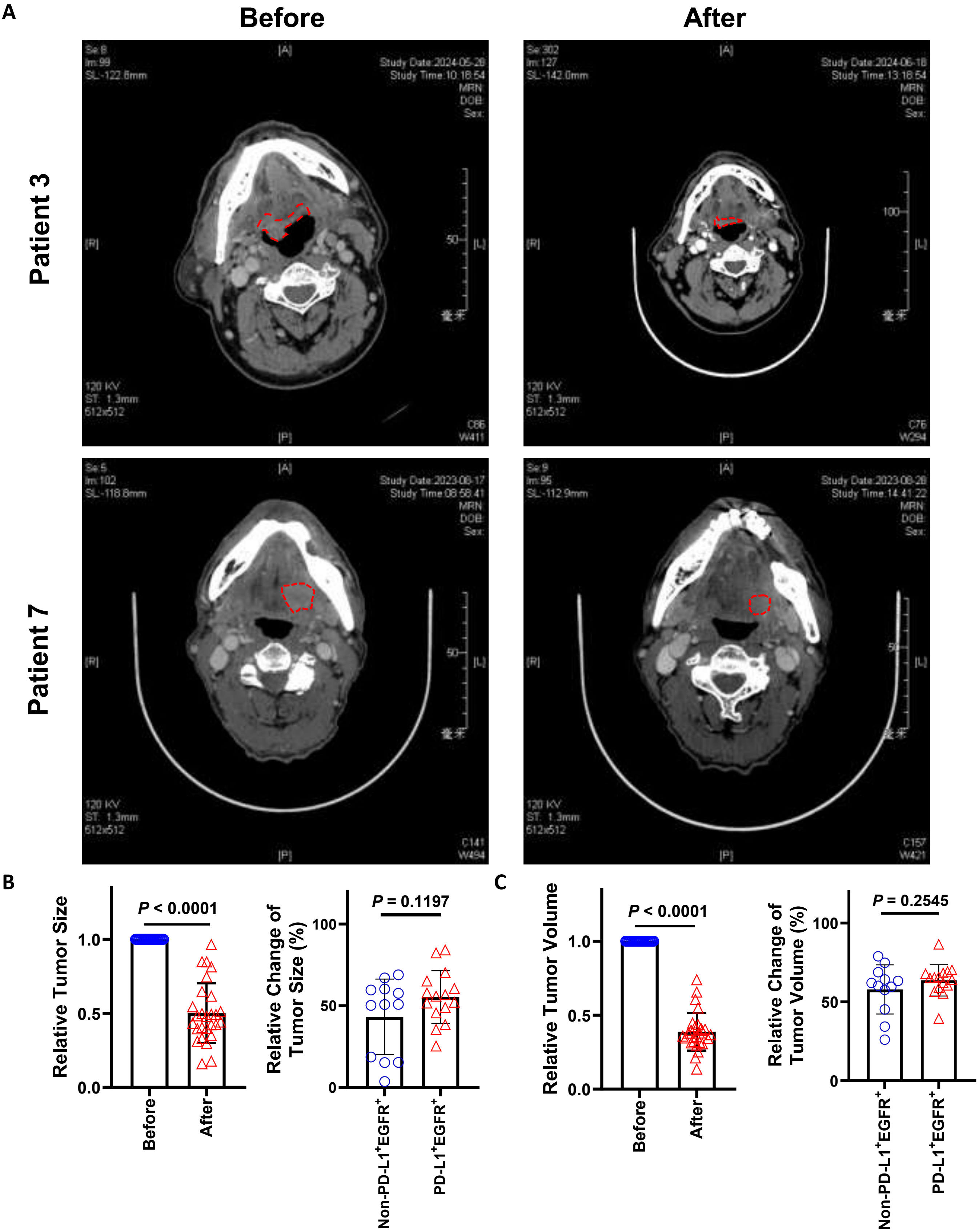

Fig. S1 Anti-PD-1 and EGFR-based Neoadjuvant Therapy Significantly Reduces Tumor Size of HNSCC

(A) Enhanced CT showing the tumor size of two HNSCC patients before (left) and after (right) receiving one course of anti-PD-1 and EGFR-based neoadjuvant therapy.

(B) Bar plots showing the relative tumor size (left) and relative change of tumor size (right) between PD-L1^{+} EGFR^{+} and non-PD-L1^{+} EGFR^{+} HNSCC patients receiving neoadjuvant therapy. Blue circle: non-PD-L1^{+} EGFR^{+} HNSCC patients, red triangle: PD-L1^{+} EGFR^{+} HNSCC patients.

(C) Bar plots showing the relative tumor volume (left) and relative change of tumor volume (right) between PD-L1^{+} EGFR^{+} and non-PD-L1^{+} EGFR^{+} HNSCC patients receiving neoadjuvant therapy. Blue circle: non-PD-L1^{+} EGFR^{+} HNSCC patients, red triangle: PD-L1^{+} EGFR^{+} HNSCC patients.

Fig. S2 PD-L1+ EGFR+ HNSCC Patients Exhibiting an Aggressive Invasive Pattern

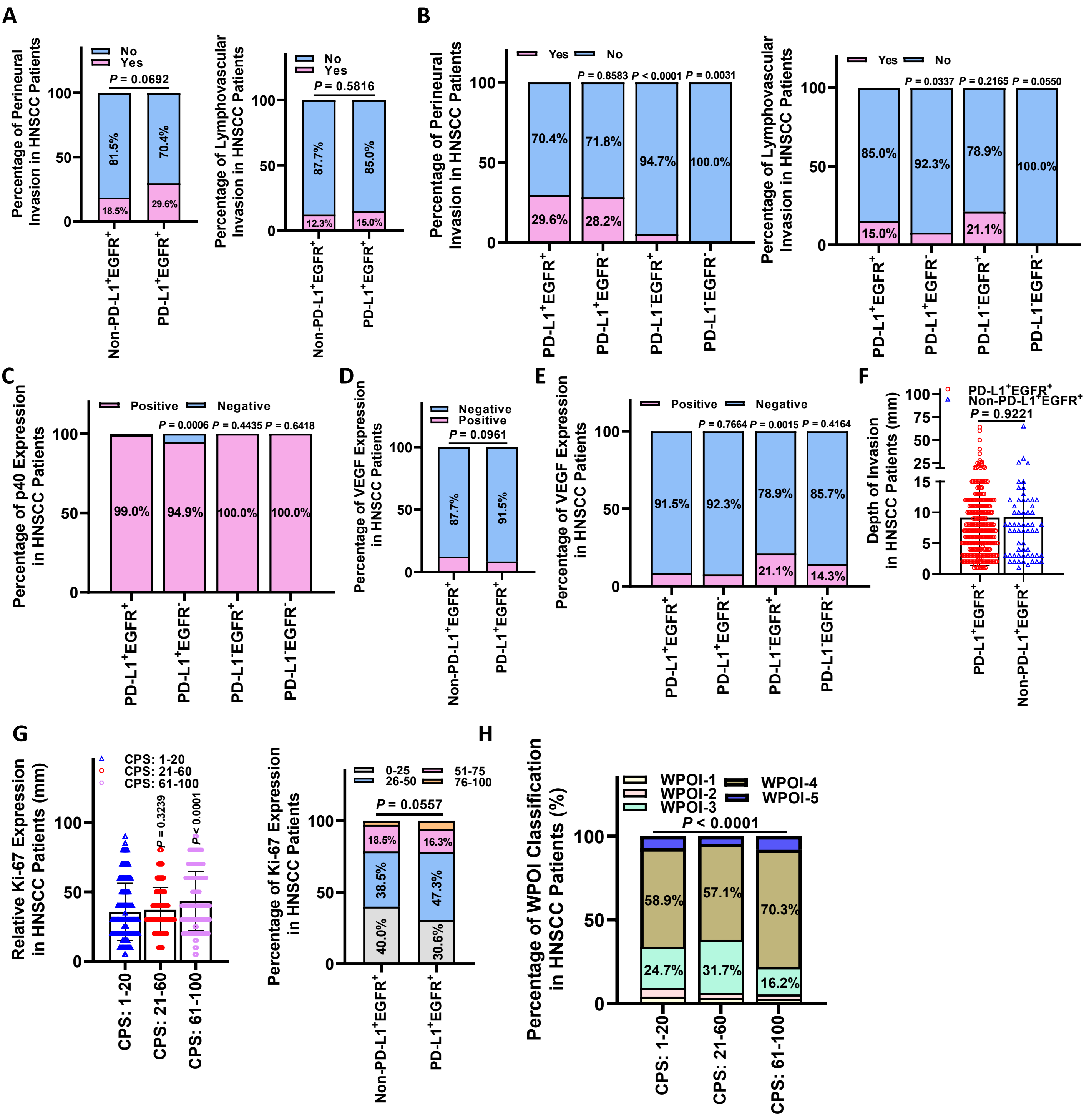

Fig. S2 PD-L1$^+$ EGFR$^+$ HNSCC Patients Exhibiting an Aggressive Invasive Pattern

A) Proportion of perineural (left) and lymphovascular invasion (right) between PD-L1$^+$ EGFR$^+$ and non-PD-L1$^+$ EGFR$^+$ HNSCC patients.

B) Proportion of perineural (left) and lymphovascular invasion (right) among the four subgroups of HNSCC patients based on CPS score and EGFR expression.

C) Proportion of p40 expression between PD-L1$^+$ EGFR$^+$ and non-PD-L1$^+$ EGFR$^+$ HNSCC patients (left), and the four subgroups of HNSCC patients (right).

D) Proportion of VEGF expression between PD-L1$^+$ EGFR$^+$ and non-PD-L1$^+$ EGFR$^+$ HNSCC patients.

E) Proportion of VEGF expression among the four subgroups of HNSCC patients.

F) DOI of PD-L1$^+$ EGFR$^+$ and non-PD-L1$^+$ EGFR$^+$ patients in our HNSCC cohort. DOI: depth of invasion.

G) Relative expression of Ki-67 among the three subgroups of PD-L1$^+$ EGFR$^+$ HNSCC patients based on the CPS score (left), and the proportion of Ki-67 expression between PD-L1$^+$ EGFR$^+$ and non-PD-L1$^+$ EGFR$^+$ HNSCC patients (right).

H) Proportion of WPOI classification between PD-L1$^+$ EGFR$^+$ and non-PD-L1$^+$ EGFR$^+$ HNSCC patients (left) and among the three subgroups of PD-L1$^+$ EGFR$^+$ HNSCC patients based on the CPS score (right). WPOI: worst pattern of invasion.

Fig. S3 CXCL11/CXCR3 Pair Enhances infiltration of pDC and Treg Cells to Restrict Anti-tumor Response

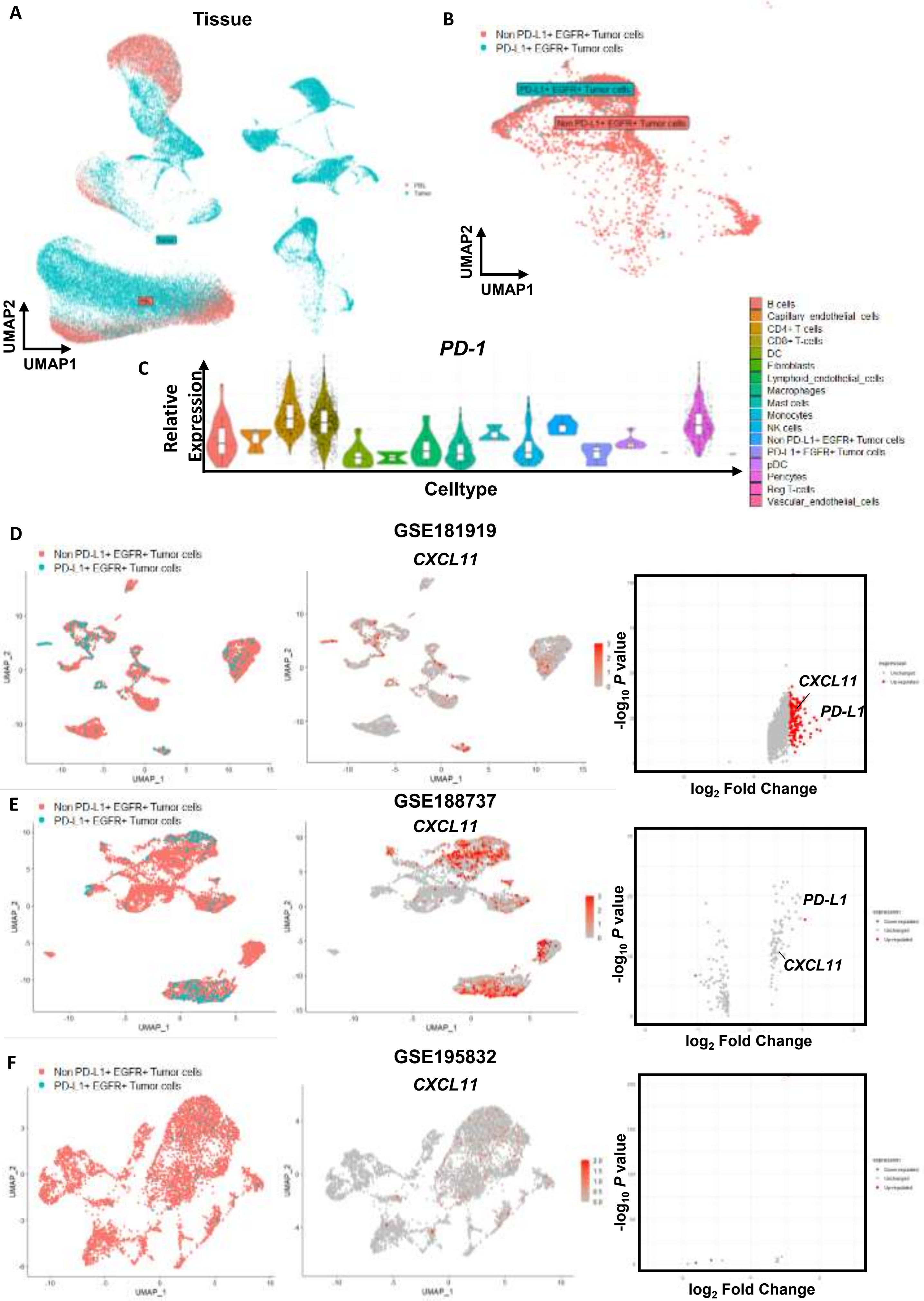

Fig. S3 CXCL11/CXCR3 Pair Enhances infiltration of pDC and Treg Cells to Restrict Anti-tumor Response

(A) UMAP plot exhibits 2 types of tissue derivation in the public scRNA-seq data (GSE164690) of HPV-negative HNSCC.

(B) UMAP plot of secondary clustering analysis for the identification of PD-L1$^+$ EGFR$^+$ and non-PD-L1$^+$ EGFR$^+$ tumor cells in HPV-negative HNSCC.

(C) Violin plots showing the relative expression level *PD-1* among all cells in the TME of HPV-negative HNSCC.

(D) UMAP plot of PD-L1$^+$ EGFR$^+$ and non-PD-L1$^+$ EGFR$^+$ tumor cells (left), expression of *CXCL11* (medium) in public HNSCC scRNA-seq dataset (GSE181919). Volcano plot (right) showing the DEGs between PD-L1$^+$ EGFR$^+$ and non-PD-L1$^+$ EGFR$^+$ tumor cells.

(E) UMAP plot of PD-L1$^+$ EGFR$^+$ and non-PD-L1$^+$ EGFR$^+$ tumor cells (left), expression of *CXCL11* (medium) in public HNSCC scRNA-seq dataset (GSE188737). Volcano plot (right) showing the DEGs between PD-L1$^+$ EGFR$^+$ and non-PD-L1$^+$ EGFR$^+$ tumor cells.

(F) UMAP plot of PD-L1$^+$ EGFR$^+$ and non-PD-L1$^+$ EGFR$^+$ tumor cells (left), expression of *CXCL11* (medium) in public HNSCC scRNA-seq dataset (GSE195832). Volcano plot (right) showing the DEGs between PD-L1$^+$ EGFR$^+$ and non-PD-L1$^+$ EGFR$^+$ tumor cells.

Fig. S4 Potential Targets Facilitate Anti-PD-1 and EGFR-based Neoadjuvant Therapy

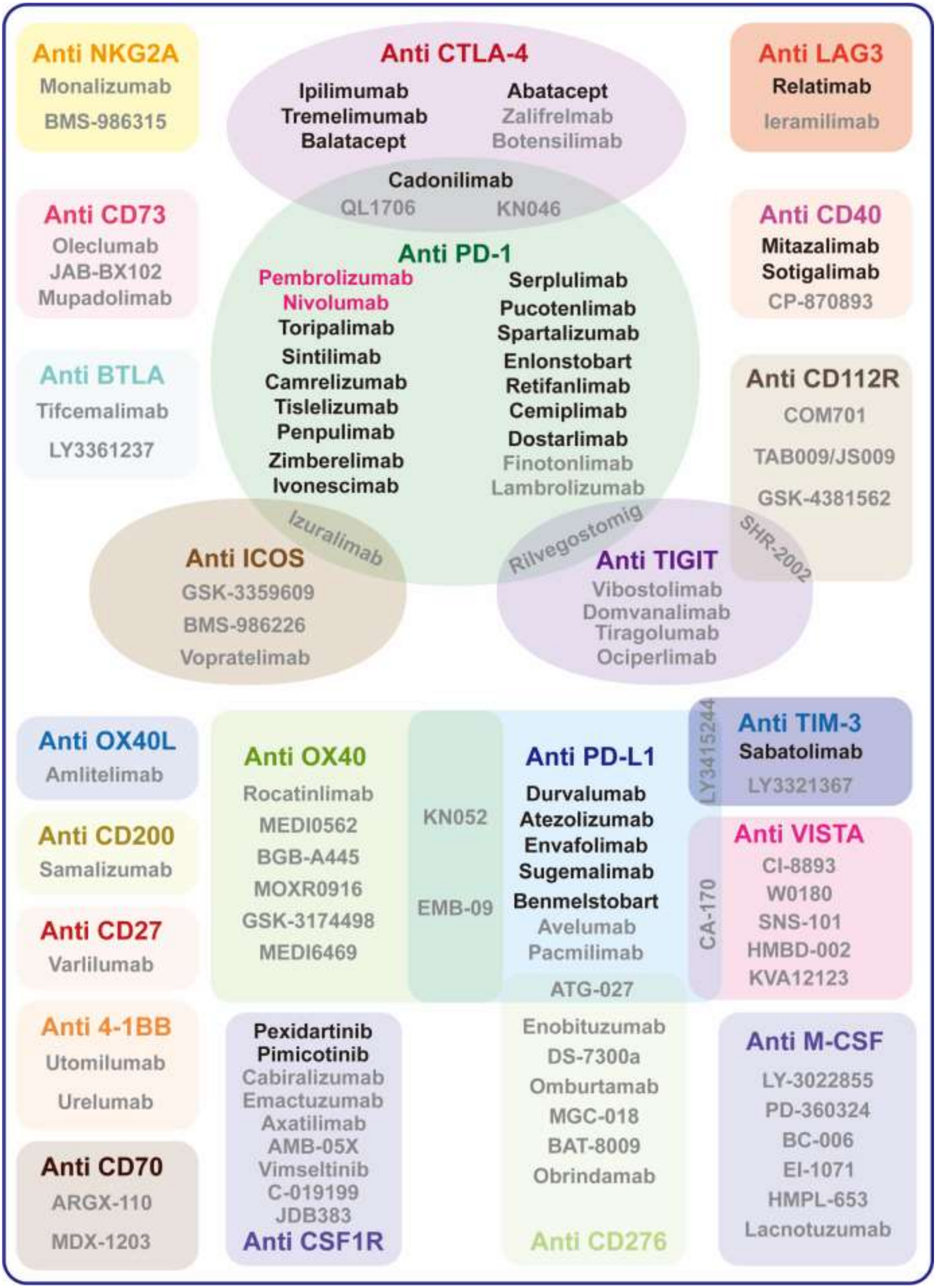

Fig. S4 Potential Targets Facilitate Anti-PD-1 and EGFR-based Neoadjuvant Therapy

Schematic diagram showing 22 immune checkpoints with inhibitors or monoclonal antibodies on clinical trials or on listed. Pembrolizumab and Nivolumab (pink) are two ICIs with the indications of HNSCC clinical treatment approved by FDA. ICIs in black have obtained indications for the clinical treatment of other solid tumors. ICIs in grey undergoes clinical trials currently.

Materials & Methods

Study design

This study, in accordance to the Declaration of Helsinki, was approved by the Joint Ethics Committee of the Affiliated Hospital of Qingdao University (Qingdao, Shandong, China) (No. AHQU-MAL20210604). 1,077 patients diagnosed with HNSCC in the Affiliated Hospital of Qingdao University was included in this cohort between May 2014 and May 2024. Inclusion criteria for this study included patients with (1) primary HNSCC tumors, (2) complete pathological diagnosis with immunohistochemistry (IHC) reports, (3) sufficient follow-up information. Patients missed in the follow-up duration were excluded from this study. None of these HNSCC patients suffered distant metastasis (M1).

Clinicopathological variables

Clinicopathological information of this cohort was collected from medical records, including gender, smoking, drinking, position of tumor, tumor differentiation, clinical stage, T and N stage, depth of invasion, perineural invasion, lymphovascular invasion, PD-L1 CPS score and the expression of EGFR, p16, p40 and VEGF. HNSCC patients in this cohort was divided into two groups based on the CPS score (CPS > 1: PD-L1$^+$; CPS < 1: PD-L1$^-$) and EGFR expression (EGFR positive: EGFR$^+$; EGFR negative: EGFR$^-$). For 27 patients who underwent anti-PD-1 (Sintilimab, 200 mg) and EGFR (Nimotuzumab, 200 mg) combined therapy for one time, HNSCC patients started combined immunotherapy after diagnosed as HNSCC both by enhanced CT and biopsy. Effect of combined neoadjuvant therapy was examined by enhanced CT before surgical treatment. For statistical analysis of continuous variable, CPS score was divided into 3 groups (<1, 1-20, >20) while Ki-67 expression into 4 groups (0-25, 26-50, 51-75, 76-100).

scRNA-seq analysis

Public scRNA-seq datasets (GEO164690, GSE181919, GSE188737, GSE195832) of HNSCC were obtained from the Gene Expression Omnibus (GEO). The raw scRNA-seq datasets were preprocessed by NormalizeData and ScaleData by Seurat R package (V 5.1.0). RunPCA, FindNeighbors, FindClusters, and RunUMAP functions were performed before the cell annotation by *SingleR* R package. Tumor cells were extracted for secondary subclassification into PD-L1$^+$EGFR$^+$ and non-PD-L1$^+$EGFR$^+$ groups. FindMarker function was conducted to obtain differentially expressed genes (DEGs) of the two groups, with avg

$|log2FC| > 1$ and q value < 0.01.

Statistical analysis

RNA sequencing (RNA-seq) data and prognosis information of HNSCC patients was obtained from the The Cancer Genome Atlas (TCGA) program (https://www.cancer.gov/ccg/research/genome-sequencing/tcga). GraphPad Prism (Version 8.0) was used for to analyze the parameter data via Student's *t* test and Pearson's *chi*-square test. Log-rank (Mantel-Cox) test was applied for the prognostic analysis of HNSSS patients. A *P* value less than 0.05 was recognized as statistically significance.
